# Methicillin-resistant *Staphylococcus aureus* ST398 in Humans and Animals, Central Europe

**DOI:** 10.3201/eid1302.060924

**Published:** 2007-02

**Authors:** Wolfgang Witte, Birgit Strommenger, Christian Stanek, Christiane Cuny

**Affiliations:** * Robert Koch Institute, Wernigerode, Germany; † Veterinary University, Vienna, Austria

**Keywords:** MRSA, host specificity, *spa* typing, multilocus ST398, central Europe, research

## Abstract

Methicillin-resistant *Staphylococcus aureus* of clonal lineage ST398 that exhibits related *spa* types and contains SCC*mec* elements of types IVa or V has been isolated from colonized and infected humans and companion animals (e.g., dog, pig, horse) in Germany and Austria. Of particular concern is the association of these cases with cases of nosocomial ventilator-associated pneumonia.

## Abstract 2

Isolates found in persons and animals in Germany and Austria show a genetic relationship.

Methicillin-resistant *Staphylococcus aureus* (MRSA) has become an infection control problem in hospitals worldwide, mainly associated with intrahospital and interhospital dissemination of particular epidemic clonal lineages of the *S. aureus* population (hMRSA; [*1*]). MRSA primarily associated with healthcare facilities may also be disseminated to the community through colonized medical staff or discharged patients. The emergence and spread of MRSA in the community during the past 5 years, independent of the healthcare setting and in the absence of typical risk factors for nosocomial MRSA infections, are matters of further concern. These community-acquired MRSA infections are less broadly resistant to antimicrobial agents than are healthcare-associated MRSA and often contain the determinants *luk*S-*luk*F, which code for Panton-Valentine leukocidin (*2*).

Even though MRSA has been known as a nosocomial pathogen for >30 years, its development in companion animals and livestock has been rare (*3*). Recent reports, however, have documented MRSA infections in animals such as horses from Canada (*4*) and Europe (*3*) and pets (*5*,*6*). Of particular interest is whether MRSA may be transmitted between animals and humans. MRSA of clonal lineage sequence type (ST) 22 is widely disseminated in human hospitals in the United Kingdom and Central Europe. The demonstration of this lineage among MRSA isolates from staff and from pets in a small animal referral hospital in United Kingdom suggests transmission between humans and animals (*5*). Nasal colonization of veterinary staff with MRSA (ST8) from infections in horses in a veterinary hospital was frequently observed in Canada (*4*), and it was also recorded in an Austrian university veterinary hospital where horses were affected by MRSA of clonal lineage ST254 (*3*).

We report on molecular characterization of MRSA, from sporadic infections in humans and in various animal species, that belong to clonal lineage ST398 according to multilocus sequence typing (MLST). These isolates were further characterized by *spa*-sequence typing (repeat polymorphism of the X-region of the *spa* gene) and by PCR for grouping of staphylococcal cassette chromosome *mec* (SCC*mec*) elements, which contain the *mec*A gene and of which at least 5 basic types have been described.

## Methods

MRSA isolates from infections in humans and in animals were sent to the National Reference Center for Staphylococci at the Robert Koch Institute, Wernigerode Branch, in Germany, for typing by means of *Sma*I-macrorestriction pattern as well as *spa* typing. Selected isolates also underwent MLST. Four human isolates were grown from nasal swabs taken from the staff of a veterinary practice at Veterinary Analytical Center, Geesthacht, Germany. All isolates were primarily grown on sheep blood agar and confirmed by standard procedures as *S. aureus*. Eleven additional MRSA specimens of lineage ST398 (1 isolate per patient affected) were found among 4,370 MRSA isolates from patients with recognized infections. These isolates were identified by indigestibility of their whole cellular DNA when subjected to *Sma*I-macrorestriction analysis. Animal isolates were collected from 1 dog and 1 foal at the Veterinary Analytical Center, Geesthacht, Germany; from 1 pig at the diagnostic laboratory of the Institute for Microbiology and Infectious Diseases, School of Veterinary Medicine, Hannover, Germany; and from 2 horses at the Department of Orthopaedics, Veterinary University, Vienna, Austria.

Procedures and primers for DNA extraction and PCR detection of resistance genes were as described previously (*6*). Macrorestriction patterns were determined by using lysis of cells, deproteinization and digestion of DNA (here by *Sma*I and *Apa*I), and pulsed-field gel electrophoresis (*7*).

The polymorphic X-region of the protein A gene (*spa*) was amplified and sequenced according to the Ridom StaphType standard protocol (www.ridom.org). The resulting *spa*-types were assigned by using the Ridom StaphType software package (Ridom GmbH, Würzburg, Germany). The BURP algorithm, implemented in the most recent Ridom StaphType software version, was used for cluster analysis of *spa* types (*7*).

Primers used for MLST correspond to the protocol as described previously (*8*), with the exception of the forward primer for *tpi*; we used the sequence tpif 5′-GCATTAGCAGATTTAGGCGT. Antimicrobial susceptibility testing was performed by broth microdilution, performed according to DIN 58940, Deutsches Institut für Normung (*9*). SCC*mec* elements of types I to IV were characterized by using a PCR approach, including a combination of different PCR reactions (*6*). To demonstrate SCC*mec*-elements of type V, we used primers type VF/type VR, as described by Zhang et al. (*10*), as well primer pair *ccr*C9f 5′-CACTTAATCCATGTACACAG and *ccr*C-R (*10*).

The following set of primers was used for PCR for virulence-associated genes: *tst, sea, seb, sec, sed, see*, as described by Johnson et al. (*11*); for *luk*S*-luk*F, forward 5′-ATCATTAGGTAAAATGTCTGGACATGATCCA, reverse 5′-GCATCAAGTGTATTGGATAGCAAAAGC; for *cna*, forward 5′-CGGTTCCCCCATAAAAGTGAAG*,* reverse 5′-CCCATAGCCTTGTGGATTTG. Annealing temperature was 55° C; cyclic scheme and further conditions were as reported previously (*6*).

Specimen collection, characterization of the isolates, data processing, and exchange of data were performed within the framework of German public health activities for infection control and prevention of MRSA dissemination. Ethical approval was obtained within this framework as well.

## Results

Characteristics of the 20 MRSA isolates investigated are shown in the Table. All isolates share MLST ST398 with the allelic profile 3-35-19-2-20-26-39. Three different *spa*-types are obviously related (Figure). Types t11 and t34 may have been derived from each other by either deletion or duplication of 2 repeats; t1197 and t11 differ by a single nucleotide polymorphism. BURP analysis of these *spa*-types groups them as a separate cluster unrelated to other BURP clusters (*7*). A peculiarity of *S. aureus* of clonal lineage ST398 is the indigestibility of whole cellular DNA by restriction enzyme *Sma*I. Therefore, *Sma*I macrorestriction patterns generate only 1 large fragment because of protection by a novel DNA methylation enzyme (*12*). We also found poor digestion by the isoschizomeric enzyme *Xma*I. However, digestion by enzyme *Apa*I generated similar fragment patterns that differed at most by 3 fragments independent of *spa* types.

**Figure:**
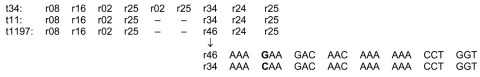
Repeats of the X-region in methicillin-resistant *Staphylococcus aureus* A of clonal lineage ST398.

The 2 horse isolates from the Vienna veterinary university contained SCC*mec* elements of group IVa. For all other isolates investigated, PCR indicated SCC*mec*V. These findings suggest that MRSA of ST398 from horses are unrelated to the other isolates and probably have evolved independently by acquisition of a different SCC*mec* element.

In addition to *mec*A, all investigated isolates contained *tet*M; isolates from animals and humans from Lower Saxony also contained *erm*A. The nosocomial human and horse isolates contained *erm*C; in the horse isolates, aph2″-aac6′–mediating aminoglycoside resistance was demonstrated. PCR was negative for virulence-associated genes and for *luk*S-*luk*F (coding for Panton-Valentine leukocidin), *tst*, *sea*, *seb*, *sec*, and *sed*, as well as for *cna* (collagen-binding protein).

## Discussion

Isolates of clonal lineage ST398 seem not to be frequently represented among the *S. aureus* population. They were not recorded by Grundmann et al. (*13*) among a population sample of nasal colonizers in the Nottingham area in the United Kingdom and were not found among 108 isolates from carriers in a rural territory in northern Germany (S. Holtfreter et al., unpub. data). Only 2 notations of ST398 are found in the *S. aureus* MLST database (www.mlst.net), 1 from the Netherlands and 1 from the Cape Verde Islands.

Among 11,250 isolates of various origin (colonization and infections in hospitals as well in the community in humans from all Germany) typed from 1992 through 2003, no isolates refractory to *Sma*I macrorestrition analysis were seen. Therefore, a rather recent emergence of MRSA ST398 among humans seems likely. However, MRSA of lineage ST398 had been reported from infections in pigs and from nasal colonization in pig farmers in France (*14*). A more recent report from the Netherlands describes MRSA of ST398 (*spa* t108, which is in the same BURP cluster as t11 and t34) in pigs and in humans who had contact with pigs (*15*). A comparison of the allelic profile of ST398 by means of the MLST database does not indicate any relationship to profiles of prevalent clonal complexes of methicillin-susceptible *S. aureus* (*13*), of epidemic healthcare-associated MRSA, or of *luk*S*-luk*F–containing community-associated MRSA from Europe.

## Conclusions

MRSA exhibiting ST398 may colonize and cause infections in humans and in certain animal species such as dogs, horses, and pigs. The isolation of MRSA ST398 showing the same characteristics from a wound infection in a dog and from nasal colonization of the staff of a veterinary practice where this dog had been treated suggests that interspecies transmission may occur. The differences in *spa*-types between the isolates containing the same PCR results for SCC*mec* can be explained by a single genetic event. Because isolates taken at the same time from nasal colonization in veterinary staff of the same practice exhibit either *spa*-type t011 or t034, this difference does not justify discrimination between the two types. Of particular concern was the subsequent detection of MRSA ST398 not only in outpatients but also in inpatients with ventilator-associated pneumonia in the same hospital unit at about the same time (Table).

**Table:** Typing characteristics of methicillin-resistant *Staphylococcus aureus* of clonal lineage ST398 from animals and humans

Country/area	Carrier; type of infection	No. isolates	*spa*-type	SCC*mec* group	Resistance phenotype*	Resistance genes
Germany						
Lower Saxony†	Dog; skin infection	1	t034	V	PEN, OXA, ERY, CLI, OTE	*mec*A, *erm*A, *tet*M
	Human‡ (n = 4); nasal carriage	1 3	t011 t034	V	PEN, OXA, ERY, CLI, OTE	*mec*A, *erm*A, *tet*M
	Pig; colonization	1	t034	V	PEN, OXA, ERY, CLI, OTE	*mec*A, *erm*A, *tet*M
	Foal; sinusitis	1	t1197	V	PEN, OXA, OTE	*mec*A, *tet*M
Schleswig-Holstein	Human; infection of skin (outpatient)	1	t011		PEN, OXA, OTE	*mec*A, *tet*M
Lower Saxony†	Human; wound infection	1	t011	V	PEN, OXA, OTE, CIP	*mec*A, *tet*M
	Human; nasal colonization§	1	t034	V	PEN, OXA, ERY, CLI, OTE	*mec*A, *erm*A, *tet*M
Hesse	Human; nasal colonization§	1	t034	V	PEN, OXA, OTE	*mec*A, *erm*A, *tet*M
Saxony-Anhalt	Human; ventilator-associated pneumonia	1	t034	V	PEN, OXA, ERY, CLI, OTE	*mec*A, *erm*C, *tet*M
Saxony	Human (n = 2); ventilator-associated nosocomial pneumonia¶	2	t011	V	PEN, OXA, ERY, CLI, OTE, CIP	*mec*A, *erm*C, *tet*M
Baden-Württemberg	Human (n = 4); ventilator-associated nosocomial pneumonia#	4	t011	V	PEN, OXA, GEN, ERY, CLI, OTE	*mec*A, *erm*C, *tet*M
Austria						
Vienna	Horse (n = 2); wound infection	2	t011	IVa	PEN, OXA, GEN, ERY, CLI, OTE	*mec*A, *erm*C, *tet*M, *aph2"-aac6'*

*PEN, penicillin; GEN, gentamicin; ERY, erythromycin; CLI, clindamycin; OTE, oxytetracycline; CIP, ciprofloxacin. †Two Lower Saxony regions are listed separately because the isolates originated from different locations within the region and, although belonging to the same clonal lineage, exhibited different spa-sequence types and resistance traits with regard to ermA and erythromycin resistance. ‡Staff members who worked in the practice where the dog was treated. §Colonization detected upon hospital admission.. ¶Infections at the same ward within a 2-week period. #Infections at the same ward within a 10-day period.

Future recording of MRSA ST398 from infected and colonized humans (especially when detected by screening at admission to hospitals) will require a thorough analysis with respect to association with animals and routes of transmission. Tracing MRSA carriers among contacts should also include pet animals, horses, and other livestock. Because of the time and labor needed to complete MLST, *spa*-typing combined with BURP analysis of types is an efficient tool for recognizing this clonal lineage. Furthermore, detection of MRSA by appropriate methods should be implemented into antimicrobial resistance surveillance programs in veterinary medicine.
